# A Femtosecond Electron‐Based Versatile Microscopy for Visualizing Carrier Dynamics in Semiconductors Across Spatiotemporal and Energetic Domains

**DOI:** 10.1002/advs.202400633

**Published:** 2024-06-18

**Authors:** Yaqing Zhang, Xiang Chen, Yaocheng Yu, Yue Huang, Moxi Qiu, Fang Liu, Min Feng, Cuntao Gao, Shibing Deng, Xuewen Fu

**Affiliations:** ^1^ Ultrafast Electron Microscopy Laboratory MOE Key Laboratory of Weak‐Light Nonlinear Photonics School of Physics Nankai University Tianjin 300071 China; ^2^ School of Materials Science and Engineering Smart Sensing Interdisciplinary Science Center Nankai University Tianjin 300350 China

**Keywords:** bulk carriers, scanning ultrafast electron microscopy, surface carriers, time‐resolved cathodoluminescence, ultrafast carrier dynamics

## Abstract

Carrier dynamics detection in different dimensions (space, time, and energy) with high resolutions plays a pivotal role in the development of modern semiconductor devices, especially in low‐dimensional, high‐speed, and ultrasensitive devices. Here, a femtosecond electron‐based versatile microscopy is reported that combines scanning ultrafast electron microscopy (SUEM) imaging and time‐resolved cathodoluminescence (TRCL) detection, which allows for visualizing and decoupling different dynamic processes of carriers involved in surface and bulk in semiconductors with unprecedented spatiotemporal and energetic resolutions. The achieved spatial resolution is better than 10 nm, and the temporal resolutions for SUEM imaging and TRCL detection are ≈500 fs and ≈4.5 ps, respectively, representing state‐of‐the‐art performance. To demonstrate its unique capability, the surface and bulk carrier dynamics involved in n‐type gallium arsenide (GaAs) are directly tracked and distinguished. It is revealed, in real time and space, that hot carrier cooling, defect trapping, and interband‐/defect‐assisted radiative recombination in the energy domain result in ordinal super‐diffusion, localization, and sub‐diffusion of carriers at the surface, elucidating the crucial role of surface states on carrier dynamics. The study not only gives a comprehensive physical picture of carrier dynamics in GaAs, but also provides a powerful platform for exploring complex carrier dynamics in semiconductors for promoting their device performance.

## Graphical abstract

A femtosecond electron‐based versatile microscopy that combines scanning ultrafast electron microscopy imaging and time‐resolved cathodoluminescence detection allows for directly visualizing and decoupling complex carrier dynamics involved in surface and bulk of semiconductors with unprecedented spatiotemporal and energetic resolutions.

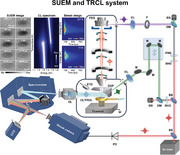

## 1 Introduction

Understanding and regulating carrier dynamics in semiconductors are critical for optimizing the performance of their photovoltaic, photocatalytic, sensing, and light‐emitting devices.^[^ ^1^, ^2^, ^3^, ^4^ ^]^ Generally, carrier dynamics in semiconductors include generation, diffusion, separation, trapping, radiative and non‐radiative recombination processes, predominantly occurring on time scales ranging from femtoseconds (fs) to picoseconds (ps) or nanoseconds (ns) and spatial scales from nanometers (nm) to micrometers (µm). These processes are intricately intercoupled in space and time, while accomplished with energy exchange in various carrier forms, which are ultimately determined by the intrinsic physical properties of the materials, such as energy band structures, surface states, and defect states.^[^ ^5^, ^6^, ^7^ ^]^ While regarding the different semiconductor functional devices, specific regulation of certain carrier dynamic processes is essential. For instance, photovoltaic solar cells^[^ ^8^ ^]^ and photocatalytic materials^[^ ^9^ ^]^ necessitate rapid separation and efficient transfer of photoexcited charge carriers at surfaces or interfaces; photodetectors^[^ ^10^ ^]^ require to improve the directional mobility of photogenerated electrons and holes under applied bias; light‐emitting diodes^[^ ^11^ ^]^ require the enhancement of radiative recombination and suppression of non‐radiative processes of carriers in an effective manner at their junction interfaces, which are usually on the nanometer or micrometer scale. Therefore, decoupling various types of carrier dynamic processes with high spatiotemporal resolution and understanding their microscopic physical mechanisms are of great importance to advance the device application of semiconductors.

Over the past few decades, a variety of time‐resolved optical techniques have been developed and employed to elucidate carrier dynamics in semiconductors, such as transient absorption microscopy (TAM)^[^ ^12^ ^]^ or spectroscopy,^[^ ^13^ ^]^ time‐resolved near‐field scanning optical microscopy (NSOM),^[^ ^14^ ^]^ time‐resolved photoluminescence (TRPL),^[^ ^15^ ^]^ time‐resolved photoemission electron microscopy (TR‐PEEM),^[^ ^16^ ^]^ etc. However, due to the limitation of optical wave diffraction and the considerable penetration depth of laser, these techniques generally tend to obtain the average carrier dynamics information in the range of several hundred nanometers or even micrometers within the materials, substantially masking the surface or interface contributions. To overcome these drawbacks and fully characterize the carrier dynamics, a probing technique with simultaneous high resolutions in multiple dimensions of time, space, and energy, as well as high surface sensitivity is highly demanded.

Recently, ultrafast electron microscopy techniques based on ultrafast pulsed electrons have flourished, opening up new horizons for the investigation of dynamic processes in materials with combined high spatial resolution of electron microscopy and high temporal resolution of ultrashort laser.^[^ ^17^, ^18^, ^19^, ^20^, ^21^ ^]^ Among them, SUEM technique that utilizes fs laser pulses for pump and synchronized fs electron pulses for probe, is a surface‐sensitive technique capable of directly imaging the spatiotemporal evolution of photogenerated charge carriers (electrons and holes) at material surfaces and interfaces.^[^ ^22^, ^23^, ^24^ ^]^ So far, applications in imaging surface/interface photocarrier dynamics in various semiconductors have demonstrated the unique advantages of the SUEM technique, such as surface carrier dynamics in silicon,^[^ ^25^ ^]^ GaAs,^[^ ^26^ ^]^ MoS_2_,^[^ ^27^ ^]^ boron arsenide,^[^ ^28^ ^]^ black phosphorus,^[^ ^29^ ^],^ etc. Nevertheless, the SUEM technique alone is not able to distinguish the complex recombination pathways of photocarriers in the energy domain, such as radiative and non‐radiative recombination processes, which are coupled in the SUEM imaging signals. Fortunately, the advent of picosecond time‐resolved cathodoluminescence (TRCL) technique, which is based on using fs or ps electron pulses for excitation of transient luminescence in the specimen, provides an energy‐sensitive means for probing the radiative recombination dynamics of non‐equilibrium carriers with high spatiotemporal resolution.^[^ ^30^, ^31^, ^32^ ^]^ Early in 1980, using an electric beam blanker to obtain pulsed electrons in SEM, Hastenrath et al. realized TRCL with ≈100 ps time resolution for determining the radiative recombination lifetime of excited carriers in GaAs.^[^ ^33^ ^]^ In 2005 and later, through the use of fs photoemission electron pulses in SEM, the temporal resolution of TRCL was improved to ≈10 ps.^[^ ^30^ ^]^ Most recently, Kim et al. have integrated TRCL into an ultrafast transmission electron microscope and revealed emitting states of the NV° centers in nanodiamonds with a local sensitivity of ≈50 nm and a temporal resolution of 100 ps.^[^ ^34^ ^]^ Therefore, TRCL detection is highly complementary to SUEM imaging in terms of experimental methodology for probing and decoupling carrier dynamics in semiconductors with high spatiotemporal resolution. Since both the realization of SUEM imaging and TRCL detection are based on ultrafast electron pulses, they could in principle share the same ultrafast photocathode inside a scanning electron microscope (SEM). Therefore, one feasible choice to achieve full characterization of carrier dynamics in semiconductors, especially the different carrier dynamic processes involved in the bulk and surfaces/interfaces in multiple dimensions of time, space, and energy with simultaneous high resolutions is to combine TRCL detection with SUEM imaging in one SEM platform.

In this work, we report the development of a novel fs electron‐based versatile microscopy with integrated SUEM imaging and TRCL detection functionalities that permits to directly image and decouple complex carrier dynamics in semiconductors in space, time, and energy dimensions with unprecedented resolutions. The spatial resolution of the system is better than 10 nm, and the temporal resolutions for the SUEM imaging and the TRCL detection are ≈500 fs and ≈4.5 ps, respectively, representing state‐of‐the‐art performance. As a first application of this microscopy platform, and to demonstrate the unique capabilities, we utilize multiple detection modes to fully understand and decouple the intricate carrier dynamics involved in the surface and bulk of n‐type GaAs, one of the most widely used III‐V compound semiconductors in photodetectors,^[^ ^35^ ^]^ light emitters^[^ ^36^ ^]^ and camera sensors.^[^ ^37^ ^]^ Particularly, we revealed, in real time and space, that the hot carrier cooling, defect trapping, and interband‐/defect‐assisted radiative recombination correspond to a super‐diffusion, localization, and sub‐diffusion processes of the surface carriers, unraveling the important impact of the surface trapping states on the carrier dynamics in semiconductors. Predictably, this novel fs electron‐based versatile microscopy with a combination of fs SUEM imaging and picosecond TRCL detection provides a new powerful tool for fully exploring the complex ultrafast carrier dynamics in optoelectronic materials with unprecedented spatial, temporal, and energetic resolutions.

## 2 Results and Discussion

### 2.1 Construction of Femtosecond Electron‐Based Microscopy Combining SUEM Imaging and TRCL Detection

**Figure 1:**
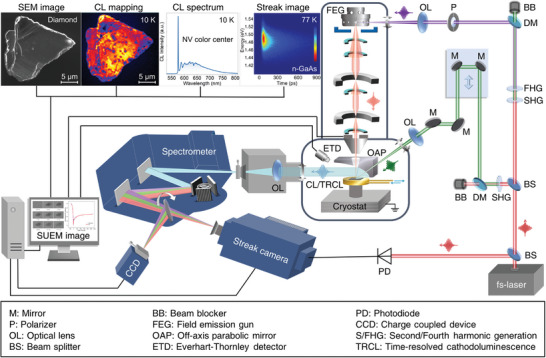
Schematic of the fs electron‐based microscopy combining SUEM imaging and TRCL detection. The entire microscopy platform includes a modified SEM with the integration of an ultrafast fs optical system, a CL/TRCL signal collection and detection module, and a cryostat with controllable sample temperature (from 10 to 300 K). The representative measurement results of various operations of the microscopy platform are displayed in the top‐left inset of the schematic diagram, of which from the left to the right are the SEM image, CL mapping image, and CL spectrum of a diamond sample at 10 K, as well as a streak camera image of a n‐type GaAs sample at 77 K, respectively. The bottom‐left inset shows a set of typical SUEM images and a corresponding dynamic curve of the n‐type GaAs. The full names of the abbreviations for the components in the schematic are shown at the bottom of the schematic.

The conceptual design of our home‐made fs electron‐based microscopy platform combining the SUEM imaging and TRCL detection is schematically presented in **Figure** **1**, which outlines the innovative combination of a SEM (Quattro S, Thermofisher) with modified thermionic Schottky field emission gun (FEG) and sample chamber, a fs laser system, and a TRCL signal collection and detection module. Specifically, the SUEM imaging and TRCL detection modules share the same fiber fs laser (YactoFiber‐FL‐50‐200, Yacto Technology Co.), which delivers a 1030 nm infrared (IR) fs laser beam with a pulse duration of ≈250 fs and repetition rate ranging from 500 kHz to 50 MHz. The initial IR beam is divided into two parts by a beam splitter (BS), one of which is frequency doubled and quadrupled with a set of two nonlinear *β*‐BaB_2_O_4_ (BBO) crystals to generate second harmonic pulses (515 nm) and fourth harmonic UV pulses (258 nm) for pumping the sample inside the SEM chamber and for focusing tightly onto the cooled Schottky field emission tip (ZrOx/W(100)) inside the FEG gun by side illumination to generate fs electron pulses, respectively; the other one is directed to enter a silicon photodetector to generate synchronous electric pulses to trigger the streak camera (C10910, Hamamatsu) for TRCL detection. Note that the fs electron pulse plays different roles in the SUEM imaging and TRCL detection. For the SUEM imaging based on the photon‐pump/electron‐probe principle, the 515 nm fs laser pulse is focused (with a diameter of ≈63 µm, see Figure S1, Supporting Information) and incident on the specimen surface to initiate the carrier dynamics, while the fs electron pulse (accelerated to 30 kV) serving as a probe beam is focused and raster‐scanned over the excitation region. Subsequently, the emitted transient secondary electrons (SEs) carrying the dynamical information of the excited carriers are collected by a positively biased Everhart–Thornley detector (ETD) to generate a SEM image in the photoemission pulsed electron mode. A computer‐controlled optical delay stage is applied to precisely control the time delay between the pump laser pulse and probe electron pulse, which defines the time axis of the recorded time‐resolved SEM images with a variable delay time ranging from −0.6 to 7.0 ns. Finally, the one far from time zero (before laser excitation) is subtracted from the time‐resolved SEM images at different delay times to obtain the “SUEM images”, also known as “contrast images” or “difference images”, which can directly reflect the spatiotemporal evolution of the photocarrier dynamics.^[^ ^38^, ^39^ ^]^ The repetition rate of fs laser used in the SUEM imaging mode is tunable from 1 to 10 MHz. Unless specified, the repetition rate is 5 MHz in the SUEM measurements. For the TRCL detection mode, the focused high‐energy fs electron pulse is used as an excitation source to bombard the specimen to generate excited carriers and the subsequent transient CL. The TRCL signal is then collected by an off‐axis parabolic mirror (OAP) and guided out of the SEM chamber to be focused into a grating spectrometer (iHR 320, HORIBA) by an optical lens (OL). To ensure optimal collection efficiency, the focused point of the OAP is situated at ≈9.6 mm below the SEM pole piece. After the spectrometer, the dispersed transient CL signal is ultimately received by a streak camera to acquire the time‐ and energy‐resolved CL or by a charge‐coupled device (CCD) to record the time‐integrated energy‐resolved CL spectrum or energy‐filtered CL imaging. Note that in the TRCL detection mode, the repetition rate of the fs laser is switched to 50 MHz to meet the synchronization requirement of the streak camera. Moreover, to further improve the experimental capability of this multimode microscopy platform, a cryostat that enables the sample temperature controllable from 10 to 300 K has been equipped, which makes it possible to study dynamic processes at different low temperatures, such as the Bose‐Einstein condensation of excitons,^[^ ^40^ ^]^ the charge density wave dynamics in phase transitions,^[^ ^41^ ^]^ and even the cooper pair dynamics in superconductors.^[^ ^42^ ^]^

In addition to the full characterization of carrier dynamics in semiconductors by the SUEM imaging and TRCL detection (shown by the typical SUEM and streak camera images in the insets of Figure 1), which will be elaborated later in the following parts, another advantage of this fs electron‐based microscopy platform is that it is easily switchable between the photoemission fs electron beam mode and the conventional continuous field emission electron beam mode. Thus, it also keeps the high spatial‐resolution SEM imaging and CL mapping capabilities under the field emission mode for providing the detailed morphology of the specimen and revealing the localized distribution of the luminescent defects through their emission photon energy and intensity. For example, the top inset of Figure 1 shows the representative SEM image, time‐integrated CL spectrum, and corresponding photon energy‐filtered CL map of a diamond sample at 10 K using the field emission mode, providing a clear identification of the single photon emission at ≈575 nm corresponding to the nitrogen‐vacancy (NV^0^) zero‐phonon line (ZLP) in the diamond^[^ ^43^ ^]^ and the spatial distribution of the NV^0^ defects. Therefore, it is a versatile electron microscopy platform that enables convenient switching between multiple operation and detection modes to characterize the morphology, intrinsic/defect luminescence properties, and carrier dynamics at the same microscopic regions of the specimen, ensuring the consistency of the experimental conditions and excluding any risk of contamination and oxidation of the sample between different measurements, which are particularly beneficial for the characterization of micro/nanostructures and air‐sensitive materials.

### 2.2 Performance Characterization of Femtosecond Electron‐Based Microscopy Combining SUEM Imaging and TRCL Detection

**Figure 2:**
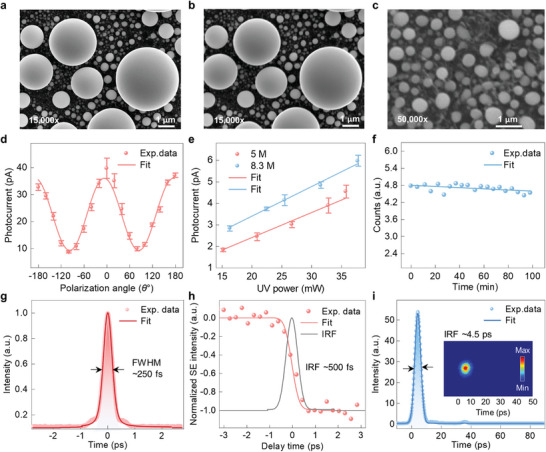
Performance characterization of the fs electron‐based microscopy combining SUEM imaging and TRCL detection. a) Typical SEM image of tin spheres on a carbon film under conventional continuous field emission mode. b,c) SEM images of tin spheres on a carbon film under photoemission pulsed electron mode at different magnifications. In both imaging modes, the tin spheres show similar edge sharpness, indicating the high imaging quality of the pulsed electron mode. d) Polarization‐dependent photocurrent of the fs electron beam under photoemission mode. The relative polarization angle between the fs laser pulse and the emission tip is well fitted with a cos^2^ *θ* function (red solid curve), where *θ* is the relative angle between the UV laser pulse polarization and the emission tip. e) Linear relationships between the UV laser power and the photoemission current at repetition rates of 5 and 8.3 MHz, respectively. f) The counts of SUEM images as a function of time, which maintains a steady and very slow linear decay over time, implying the long‐term stability of the photoemission pulsed electrons. g) Temporal profile of the initial IR laser pulse. The full width at half maximum (FWHM) of the laser pulse (fitted with the sech^2^ function) is ≈250 fs (red solid curve). h) Time‐dependent SE intensity (red balls) retrieved from the SUEM images of a p‐type GaAs single crystal. The pink solid line was fitted with an error function. The FWHM of the deconvoluted Gaussian function (grey solid curve) that represents the IRF of the SUEM imaging is ≈500 fs. i) Extracted time profile of the streak image (see insert) obtained by introducing 515 nm fs laser pulses (≈310 fs) directly into the TRCL signal detection module. Gaussian function fitting (blue solid curve) reveals that the IRF of the TRCL detection is ≈4.5 ps.

To characterize the imaging performance of the home‐made fs electron‐based microscopy platform, the SEM images of a standard sample of randomly distributed nanoscale tin spheres (with diameters ranging from 10 nm to several micrometers) on a carbon film were obtained by using a continuous field emission electron beam and a fs pulsed electron beam, respectively (**Figure** **2**a–c). Among them, the SEM image under photoemission pulsed electron mode was obtained by an integration of 128 frames with a dwell time of 300 ns at each pixel. As shown clearly in the images (Figure 2a,b) at 15 000× magnification, the sharp edges of the tin spheres under continuous field emission electron mode are comparable to the photoemission pulsed electron mode. Further quantitative analysis of the intensity profile (Figure S2, Supporting Information) shows that the rising width of the sphere's edge (≈0.11 µm) in Figure 2a is close to that (≈0.14 µm) in Figure 2b, indicating the comparable high imaging quality of the fs electron mode to that of the field emission mode. Moreover, at a higher magnification of 50 000×, the nanoscale tin spheres smaller than 10 nm are still clearly distinguishable in Figure 2c, demonstrating the high spatial resolution of the photoemission pulsed electron imaging mode. According to the spatial resolution deterministic equation β = δ/*M* (where *M* (≈50 000×) is the effective magnification and δ (≈0.3 mm) is the minimum distance distinguishable by human eye),^[^ ^44^, ^45^ ^]^ the spatial resolution of the fs electron mode can be estimated to be better than 10 nm, which is similar to the results of the previous studies.^[^ ^22^ ^]^ The good image quality and high spatial resolution reflect the excellent stability and small energy spread of the fs electron beam in our instrument.

Because of the side illumination of the UV laser pulse on the Schottky field emission tip, the polarization angle θ of the UV laser pulse relative to the emission tip is important to maximize the photoemission efficiency.^[^ ^46^ ^]^ As presented in Figure 2d, at a certain UV laser power, the photocurrent follows a cos^2^θ function of the relative polarization angle θ, which is in line with the previous report.^[^ ^47^ ^]^ Apparently, the photoemission efficiency reaches the maximum when the UV laser polarization is parallel to the emission tip, substantiating the presence of the photon electric‐field assisted emission process. Unless specified, the polarization of the UV laser pulse is fixed parallel to the emission tip in all the experiments. Figure 2e shows the relationship between the photocurrent and the UV probe laser power under two different repetition rates (measured by a Faraday cup connected to a picoammeter). Similar to the previous results,^[^ ^22^, ^47^ ^]^ both of them show a linear UV laser power dependence without any saturation, indicating that no damage to the emission tip happens within the measured laser power range. Note that the slopes of the two linear curves are nearly the same, indicating similar photoemission quantum efficiency at both 5 and 8.3 MHz, ruling out the existence of a significant thermal accumulation effect. As is well known, the space‐charge repulsion between electrons in the photoemission fs electron pulse substantially diminishes the temporal resolution, so usually the fewer electrons in each pulse, the better the temporal resolution. However, to achieve a sufficient signal‐to‐noise ratio (SNR), an appropriate brightness of the total beam current is required. Therefore, to keep a good compromise between the temporal resolution and the SNR, the SUEM imaging mode is usually operated under the UV probe laser with a repetition rate of 5 MHz and a pulse energy of ≈4 nJ. Figure 2f displays the time‐dependent total counts of the SUEM image intensity over several hours, in which the value of the total counts gently decays in a linear manner, demonstrating the excellent long‐term stability of the fs electron emission and the reliability of long‐term recording of carrier dynamics.

To examine the temporal resolutions of the SUEM imaging and TRCL detection in our home‐made fs electron‐based microscopy, we have further carried out measurements following their corresponding standard test methods. First of all, we measured the temporal intensity profiles of the initial IR and 515 nm fs laser pulses, which follow well the shape of the *sech* ^2^ function (Figure 2g and Figure S3, Supporting Information), and the retrieved full width at half maximum (FMHW) of the pulse durations for them are ≈250 and ≈310 fs, respectively. The longer pulse duration of the second harmonic pulse is due to the chirp broadening effect induced by the BBO crystal. To determine the temporal resolution of the SUEM imaging mode, we carried out SUEM imaging measurement on a freshly cleaved cross‐section of a p‐type GaAs single crystal with a probe laser pulse energy of ≈0.2 nJ. As shown by the SUEM images at different delay times in Figure S4 (Supporting Information), upon the pump pulse excitation dark contrast appears and intensifies rapidly, indicating the ultrafast evolution of the photocarrier distribution upon the laser pulse excitation. Notice that the falling edge of the extracted dynamic curve near the time zero can be well fitted using the error function 1 + *erf*[(*t* − *t*_0_)/τ] (Figure 2h), which is a step function convolved with a Gaussian function. One can retrieve the FWHM of the Gaussian function that corresponds to the instrument response function (IRF), which is ≈500 fs, demonstrating the high temporal resolution of the SUEM imaging mode. For the temporal resolution of the TRCL detection mode, since the time response of the streak camera is <2.0 ps and the duration of the photoemission electron pulse is <1.0 ps,^[^ ^48^ ^]^ it is mainly limited by the time jitter between the excitation electron pulse and the detection system. We thereby introduced the short second harmonic laser pulses (≈310 fs) directly into the TRCL collection module to acquire a streak camera image to evaluate the temporal resolution. As shown in Figure 2i, the FWHM of the intensity temporal profile extracted from the streak camera image (inset of Figure 2i) is ≈4.5 ps, which is significantly improved compared to the previous work (≥ 10ps),^[^ ^34^, ^48^ ^]^ representing state‐of‐the‐art temporal resolution achieved hitherto in the TRCL detection. Therefore, the sub‐10 nanometer and sub‐picosecond/picosecond spatiotemporal resolution achieved in our homemade fs electron‐based microscopy platform provides a solid foundation for the joint applications of SUEM imaging and TRCL detection to explore the comprehensive carrier dynamics involved in surface/interface and bulk in semiconductors with unprecedented spatiotemporal resolution.

### 2.3 Experimental Study of Carrier Dynamics in n‐Type GaAs with Combined SUEM Imaging and TRCL Detection

To demonstrate the unique capability of the complementary SUEM imaging and TRCL detection in our home‐made fs electron‐based microscopy for imaging and decoupling the complex carrier dynamics in semiconductors, especially for distinguishing the surface and bulk carrier dynamics in real time and space, we have systematically investigated the carrier dynamics in heavily silicon‐doped n‐type GaAs, a representative III‐V compound semiconductor with a direct bandgap, high carrier mobility, and luminescence efficiency.^[^ ^49^ ^]^ The reason for choosing the GaAs as the study object is that it is one of the most widely used semiconductors in modern optoelectronic devices, such as high‐speed photodetectors,^[^ ^35^ ^]^ camera sensors,^[^ ^50^ ^]^ etc. For these GaAs‐based high‐speed and ultrasensitive optoelectronic devices, efficient regulation and control of photocarriers in the GaAs surfaces and interfaces are fundamentally crucial, such as fast separation and collection of the excited electron–hole pairs while suppressing their recombination. To this end, a comprehensive understanding of the impacts of surface/interface states arising from unsaturated dangling bonds, passivation coating layer or absorbents and bulk defects including vacancies and dopants that could form carrier trapping or recombination centers on the carrier dynamics of GaAs is highly indispensable.^[^ ^51^, ^52^ ^]^ Nevertheless, distinguishing the surface/interface carrier dynamics and bulk carrier dynamics of GaAs across spatial, temporal, and energetic domains with high resolutions has not been well experimentally established.

**Figure 3:**
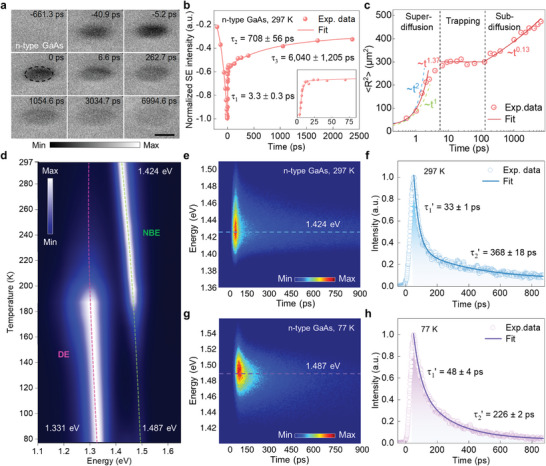
Experimental results of SUEM imaging and TRCL measurements on a heavily silicon‐doped n‐type GaAs single crystal. a) Temporal evolution of SUEM images acquired on the (100) surface of the n‐type GaAs crystal at room temperature. The pump laser fluence is ≈7.48 µJ cm^−2^. The black dashed ellipse in the SUEM image of 0 ps represents the footprint of the pump laser on the sample. The scale bar is 50 µm. b) Time‐dependent SE intensity at the center of the excitation region (red balls) extracted from the SUEM images in (a). The lifetimes were obtained by fitting the data with a ternary exponential function (solid red line). The inset shows the data and a fitted curve near the time zero. c) Temporal evolution of the second moment 〈*R* ^2^〉 of the spatial distribution of the surface carriers with the delay time plotted in log scale. The solid lines are the fitting results by power law function with different exponents, and the dashed linear (*α *= 1) and quadratic (*α *= 2) curves that correspond to normal diffusion and ballistic transport are also plotted for comparison. d) Temperature‐dependent evolution of the normalized time‐integrated CL spectra of the n‐type GaAs from 297 to 77 K. The green and pink dotted lines indicate the evolution trends of the near band emission and defect emission, respectively. e, g) Streak images acquired from the SUEM imaging measured area at 297 and 77 K, respectively. The dashed line denotes the emission energy of ∼1.424 eV and ∼1.487 eV. f, h) Corresponding decay traces of the near band emission extracted from (e) and (g), which are fitted with a biexponential function.

We used the SUEM imaging mode to study the spatiotemporal evolution of the carrier dynamics of the heavily silicon‐doped n‐type GaAs (see Experimental Section) with a native oxide layer on the (100) surface, which introduces substantial surface states and has a deterministic role on the carrier dynamics.^[^ ^53^, ^54^, ^55^ ^]^ In order to clarify the surface defects of the n‐type GaAs (100) expected in our work, we performed X‐ray photoelectron spectroscopy (XPS) measurements to investigate the chemical composition of the sample surface. As shown in Figure S5 (Supporting Information), apparent Ga_2_O_3_, As_2_O_3_, and O 1s peaks appear at binding energies of 20.4, 44.3, and 531.1 eV, respectively. Thus, the main types of defects on the GaAs (100) surface are As and Ga oxides, which is consistent with the previous studies.^[^ ^56^ ^]^ Then, SUEM images clearly exhibit dark contrast at both positive and negative delay times with the most dark contrast occurring at time zero (see **Figure** **3**a; Movie S1, Supporting Information). The dark contrast at negative delay times near the time zero is mainly caused by the additional energy loss mechanism under optical excitation, where the internal transient SEs induced by the fs electron pulses would encounter more inelastic scatterings from the subsequent photocarriers on their way to the surface, resulting in a decrease in the SE emission efficiency in the photoexcited region and thus the observed dark contrast, which is a common characteristic in the n‐type GaAs.^[^ ^26^, ^57^, ^58^, ^59^ ^]^ Note that, the contrast change at the negative delay times has no impact on that of the positive times and in the following discussion we mainly focus on the dynamics at the positive delay times. The evolution of the dark contrast at the positive delay times reflects the spatiotemporal evolution of the excited holes (minority carriers) population at the sample surface after the pulse excitation. The physical picture can be simplified as follows: 1) the surface defects generate electronic states near the Fermi level within the bandgap and cause a bending upwards of the surface energy band (Figure S6, Supporting Information), resulting in the formation of a vertical, out‐of‐plane built‐in electric field pointing from the surface to the vacuum in the surface depletion region^[^ ^60^, ^61^ ^]^; 2) upon the laser pulse excitation, the photoexcited electron‐hole pairs are spatially separated by the built‐in electric field with the holes migrating to the surface while the electrons migrating to the bulk beneath the surface; 3) meanwhile, the surface states of the n‐type GaAs serving as hole trapping centers gradually capture the holes, forming a net accumulation of holes at the surface region. These excess holes would suppress the SE emission efficiency near the surface and produce the dark contrast in the SUEM images at positive delay times.^[^ ^62^, ^63^ ^]^ Therefore, the spatiotemporal evolution of the relative SE intensity (dark contrast) in the SUEM images at positive delay times directly reflects the dynamic process of the excited holes on the surface.

To quantitatively analyze the dynamics of the surface carriers, we extracted the relative SE intensity of each SUEM image by integrating the dark contrast region and plotted them as a function of delay time in Figure 3b. After the SE intensity reaches the minimum at the time zero, it shows a rapid recovery process within several picoseconds to ≈45% of the minimum value (inset of Figure 3b), and then experiences a slower recovery process followed by a long‐lived recovery process. Note that the SE intensity does not return back to the initial value even after several nanoseconds (the whole measured delay time range). The dynamical curve can be well fitted by a ternary exponential function (Figure 3b), and the three retrieved time constants τ_1_, τ_2_, and τ_3_ are ≈3.3 ± 0.3, 708 ± 56, and 6040 ± 1,205 ps, respectively. According to the previous studies by other ultrafast optical techniques,^[^ ^63^, ^64^, ^65^, ^66^, ^67^ ^]^ the fast decay component (τ_1_) corresponds to the hot carrier cooling process, in which the excited hot carriers cool down rapidly through the phonon scattering process within a few picoseconds; the slower decay process (τ_2_) is mainly due to the interband recombination of the thermally equilibrated carriers; and the long‐lived decay component (τ_3_) is related to the recombination of the trapped carriers through the defect states. Note that the dark contrast evolution observed in the SUEM imaging results directly reveals that the hot carriers and trapped carriers involved in the three different dynamical processes are mainly the excited holes, which are usually hard to be identified by other conventional ultrafast optical spectroscopy techniques. As a comparison, we also performed SUEM imaging measurements on a freshly cleaved cross‐section of the n‐type GaAs crystal. As shown by the time‐resolved SUEM images in Figure S7a (Supporting Information), a similar dark contrast evolution was observed (see also Movie S2, Supporting Information). However, there is no apparent long‐lived decay component (τ_3_) in the dynamic curve (Figure S7b, Supporting Information) due to the much fewer surface states or trapping centers on the freshly cleaved surface. Therefore, the surface states play a crucial role in the surface carrier dynamics of the n‐type GaAs.

To reveal the spatial transport dynamics of the excited carriers on the surface of the n‐type GaAs, we further calculated the second moment 〈*R* ^2^〉 of the hole distribution by analyzing the SUEM images (see Experimental Section). The corresponding temporal evolution of the second moment 〈*R* ^2^〉 is displayed in Figure 3c, in which three distinct transport regimes can be clearly identified. Interestingly, the second moment of all the three regimes can be well fitted by a power law function of 〈*R* ^2^〉∝*t* ^α^ with various exponents. In the initial time regime from 0 to ≈4 ps, the retrieved exponent α is ∼1.37 (1 < α < 2), indicating that the initial transport process is corresponding to a super‐diffusion behavior.^[^ ^63^, ^68^ ^]^ For comparison, the linear (α = 1) and quadratic (α = 2) curves that correspond to normal diffusion and ballistic transport are also plotted in Figure 3c with green and cyan dashed lines, respectively. The effective diffusion coefficient of the hot holes was extracted to be ∼40000 cm^2^/s through fitting on the super‐diffusion region, which is much higher than that (200∼900 cm^2^ s^−1^) at room temperature reported in the previous studies.^[^ ^69^ ^]^ This super‐diffusion is caused by the rapid expansion of the hot holes in their initial hot carrier cooling process, which is typically accompanied by a much larger effective diffusivity than that of the normal diffusion process. The similar effect has also been observed in Si and BAs with SUEM measurements.^[^ ^25^, ^28^ ^]^ Subsequently, a transition of the dynamic process takes place: the growth of 〈*R* ^2^〉 slows down from ≈4 to ≈7 ps, and then it stops rising and keeps nearly constant until ≈130 ps, which means the corresponding exponent α is about 0. Such a novel behavior is mainly attributed to the hole‐trapping process resulting in localization.^[^ ^63^ ^]^ In this dynamical regime, the excited holes that migrate to the surface are gradually captured by the trapping centers on the surface and localized until all the trapping centers are fully occupied. After that, a slow diffusion process develops and presents a significant sublinear increase over a long period of duration, for which the retrieved exponent α is ∼0.13, indicating a restricted diffusion of the holes.^[^ ^70^, ^71^ ^]^ This long‐lived slow diffusion behavior can be recognized as a sub‐diffusion (α < 1) process associated with the surface trapping states. Therefore, the SUEM imaging provides a unique method for directly visualizing the surface carrier transport dynamics.

To further decouple the different carrier recombination pathways in the n‐type GaAs in conjunction with the spectral information in the energy domain, we then turned to the TRCL mode to collect TRCL and time‐integrated CL spectra at variable temperatures. As shown in Figure 3d, the time‐integrated CL spectrum at room temperature (297 K) is primarily dominated by the near band emission (NBE) peak at ≈1.424 eV, which features a slight shape asymmetry with a long tail at the low‐energy side (Figure S8, Supporting Information). Such a long tail originates from the emission of the defect trapping states, for which the luminescence is very weak at room temperature due to thermal excitation. With the temperature reduced to 77 K, the NBE gradually exhibits a blue shift to ≈1.487 eV, meanwhile, the peak centered at ≈1.331 eV becomes the prominent emission, which corresponds to the arsenic‐related antisite defect emission (DE) through trapping of the photoexcited holes by the defect states.^[^ ^72^ ^]^ To verify this DE process, we further conducted CL spectrum measurements with continuous electron beam excitation under different electron currents at 77 K (Figure S9, Supporting Information). Indeed, as the excitation current increases the intensity of the NBE gradually strengthens and finally surpasses that of the DE at a certain excitation current due to the saturation of the defect state filling. Then, we captured the TRCL signal using the streak camera at 297 K (Figure 3e). The extracted decay trace of the NBE can be well fitted with a bi‐exponential function (Figure 3f), revealing two distinct lifetime components. The short one ${\tau_{1}}^{\prime }$ (33 ± 1 ps) is associated with the hole‐trapping process by the defect states, whereas the longer one ${\tau_{2}}^{\prime }$ (368 ± 18 ps) mainly corresponds to the interband‐free carrier transition.^[^ ^73^, ^74^ ^]^ At 77 K, the short lifetime of the TRCL signal (Figure 3g) prolongs to 48 ± 4 ps (Figure 3h), while the long lifetime decreases to 226 ± 2 ps. This temperature‐dependent result implies that the proportion of the defect‐trapping processes increases at lower temperatures, leading to quenching of the interband transitions.^[^ ^72^ ^]^ Moreover, we have also attempted to collect the TRCL signals from the DE, but found that the corresponding lifetime exceeded the maximum time window of the streak camera (≈3.2 ns), suggesting the presence of a long‐lived defect‐assisted radiative recombination process.

### 2.4 Comprehensive Physical Picture of the Excited Carrier Dynamics in the n‐Type GaAs

**Figure 4:**
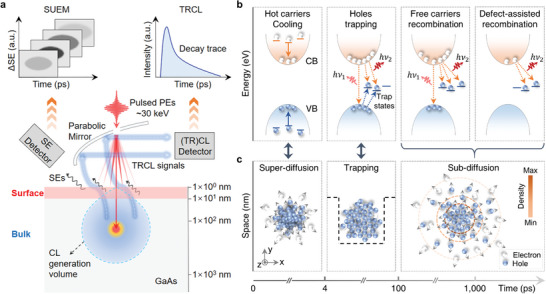
Schematic diagram of comprehensive carrier dynamics in n‐type GaAs given from a complementary perspective. a) Schematic diagram for the detection principle of the microscopy platform combining SUEM imaging and TRCL detection. When the 30 keV pulsed PEs are incident inside the GaAs, transient SEs received by the SE detector used for SUEM imaging only come from the shallow surface (<10 nm), while the CL/TRCL signals arise from a teardrop‐shaped interaction volume at a depth of several microns. b) Schematic diagram for different dynamical processes of the excited carriers in the n‐type GaAs from the perspective of its electronic band structures (energy domain) at different time scales. The short blue lines indicate the hole trap states, and the orange dashed arrows indicate the radiative recombination channels. c) Schematic diagram for the corresponding different dynamical processes of the excited carriers at the surface in real time and space.

Finally, to distinguish the surface and bulk carrier dynamics and comprehensively understand the different carrier dynamics processes involved in the n‐type GaAs, we compare and discuss the complementary natures of the SUEM imaging and TRCL detection results in terms of spatial, temporal, and energetic domains. As schematically depicted in **Figure** **4**a, considering the 30 kV accelerating voltage of the pulsed electron beam used in our measurements, when the pulsed primary electrons (PEs) bombard the GaAs surface, the internal SEs undergo massive both elastic and inelastic scatterings, resulting in a microscale teardrop‐shaped interaction volume beneath the surface (see the Monte Carlo simulation in Figure S10a, Supporting Information), in which the excited carriers produce the transient CL signals obtained in the experiment. Therefore, the TRCL result mainly reflects the ensemble‐averaged carrier dynamics of the beneath bulk region on the orders of several microns (Figure S10b, Supporting Information). In contrast, since the transient SEs can only escape from the shallow surface (<10 nm) into the vacuum, the corresponding carrier dynamics reflected by the SUEM imaging result is mainly dominated by the surface carriers or surface states.^[^ ^75^ ^]^ Therefore, by switching the two ultrafast detection modes and combining contrastive analysis of the SUEM imaging (up‐left inset of Figure 4a) and TRCL detection (up‐right inset of Figure 4a) results, it could enable distinguishing or decoupling the complicated carrier dynamics involved in both the surface and the beneath bulk region at the same microscopy area of the specimen in real time and space, including transport, defect trapping, and radiative/non‐radiative recombination processes.

Based on the analysis of the SUEM imaging and TRCL detection results, the comprehensive physical picture of the excited carrier dynamics in the measured region of the n‐type GaAs can be schematically depicted in Figure 4b,c. Upon the laser pulse excitation, the initial rapid cooling process of the hot holes accompanied by a super‐diffusion behavior on the surface within the initial 4 ps (see first panels of Figure 4b,c), which can be directly observed by the SUEM imaging in real time and space, while this process is missed in the TRCL result due to its non‐radiative nature. In the immediately following about 4–100 ps, the hole trapping process (blue dashed arrows) as well as the interband (*h*ν_1_) and defect‐assisted (*h*ν_2_) radiative recombination of the carriers predominantly occur (see second panels of Figure 4b,c). During this trapping process, the holes are gradually captured and localized by the defect states near the valence band and their spatial distribution stops expanding, namely, no apparent transverse hole transport occurs until the trapping states are fully filled, corresponding to the plateau period of the 〈*R* ^2^〉 observed by the SUEM imaging in Figure 3c. The extracted short lifetime component (33 ± 1 ps) from the TRCL result represents the decay of the interband (*h*ν_1_) radiative recombination during the hole‐trapping process. Intriguingly, the duration of the hole‐trapping process on the surface revealed by the SUEM imaging result is remarkably longer than that of the beneath bulk region revealed by the TRCL results. The reason is that there are many more trapping states on the surface than the beneath bulk region. Thus, the surface states have a great impact on the carrier dynamics. Following the hole trapping, the carriers then exhibit a sub‐diffusion behavior on the surface (last panel of Figure 4c), corresponding to several different radiative decay dynamics (third and last panels of Figure 4b). Within the delay time range of about 100–1000 ps, the interband radiative recombination process dominates. In contrast, the extracted lifetime of this process from the TRCL result is 368 ± 18 ps (Figure 3f), which is significantly shorter than that (708 ± 56 ps) obtained from the SUEM images. This discrepancy mainly arises from the different probing depths of the SUEM imaging and TRCL detection. The SUEM imaging mainly probes the carrier information near the surface, where a surface band bending effect induced by the surface states results in a surface depletion region, in which the built‐in electric field drives the excited holes toward the surface while retaining electrons beneath bulk, hindering the carrier recombination.^[^ ^76^ ^]^ Moreover, the surface state trapping effect also inhibits the carrier recombination process. While TRCL detection mainly probes carrier information in the beneath bulk, away from the depletion region, where the carrier recombination process is less inhibited. Finally, on a time scale longer than 1000 ps, the carrier dynamics is dominated by the long‐lived defect‐assisted recombination process (last panel of Figure 4b). Note that the non‐radiative recombination via deep levels in GaAs may also contribute to this process, such as the Shockley–Read–Hall (SRH) recombination.^[^ ^77^, ^78^ ^]^ Therefore, with combined SUEM imaging and TRCL measurements, the surface carrier and the beneath bulk carrier dynamics can be well distinguished, and the crucial role of the surface states and bulk defects on the carrier dynamics can be directly revealed in real time and space.

## 3 Conclusion

In summary, we have successfully developed a fs electron‐based versatile microscopy combining SUEM imaging and TRCL detection functionalities, which enables direct imaging of spatiotemporal evolution of surface carrier dynamics and decoupling surface and bulk carrier dynamics with different recombination pathways involved in the unprecedented nanometer spatial and sub‐picosecond temporal resolutions. Combining the SUEM imaging and TRCL detection, we have systematically unraveled the complex carrier dynamics in the n‐type GaAs semiconductor across spatial, temporal, and energetic domains. We directly distinguished the surface and bulk carrier dynamics in the n‐type GaAs and revealed, in real time and space, that the hot carrier cooling, defect trapping, and interband‐/defect‐assisted radiative recombination correspond to ordinal super‐diffusion, localization, and sub‐diffusion processes of the holes on the surface, elucidating the important role of the surface states on the carrier dynamics of semiconductors. Our work not only gives a comprehensive physical picture of the carrier dynamics in the n‐type GaAs, but also provides a powerful tool for exploration and comprehensive understanding of the surface/bulk carrier dynamics in semiconductors, which would be greatly helpful to the design and optimization of semiconductor optoelectronic devices.

## 4 Experimental Section

### Preparation of GaAs Single Crystals

The GaAs single crystals used in this work were heavily silicon‐doped n‐type and zinc‐doped p‐type GaAs, grown by Vertical Gradient Freeze (VGF) method. They were purchased from Beijing Jiaanheng Science & Technology Co., LTD and used without modifications. The resistivity and carrier density of GaAs are ≈10^−3^ Ω·cm and 10^18^ cm^−3^, respectively. Calculation of the second moment 〈*R* ^2^〉 for SUEM images.

The second moment of the hole distribution can be obtained by the equation:

(1) $$R^{2}\left(t\right)=\frac{\sum_{i,j}\left({x_{i}}^{2}+{y_{i}}^{2}\right)I\left(x_{i},y_{i},t\right)}{\sum_{i,j}I\left(x_{i},y_{i},t\right)}$$

For the SUEM difference images captured at delay time *t*, the *I*(*x_i_*,*y_i_*,*t*) denotes the intensity value of the pixel situated at the discrete surface coordinates (*x_i_*,*y_i_*), which was scanned by the fs electron probe. The center of the intensity distribution, established from the data and remaining fixed throughout the dynamics, is considered the origin for both the *x* and *y* axes in the aforementioned equation.

### CL and TRCL Measurements

CL spectra and images were measured by a HORIBA H‐CLUE system integrated with the modified SEM. To achieve the optimum spatial resolution with the best SNR, a focused continuous electron beam with a beam current of ≈3.6 pA was accelerated to 10 kV to produce CL signals in GaAs at room temperature. The resulting emitted photons were collected by a parabolic mirror and sent to a spectrometer equipped with triple turret gratings of 150, 300, and 1200 lines mm^−1^ to obtain the CL spectra. Unless otherwise specified, the 150 lines mm^−1^ grating with a slit width of 200 µm was used for the steady‐state CL measurements in this work. The CL spectra were then recorded by a thermoelectrically cooled silicon‐based CCD array (Synapse Plus) in the wavelength range from 200 to 1100 nm with a spectral resolution of about 0.5 nm pixel^−1^. The acquisition time was set to vary from a few seconds to several minutes, depending on the luminescence efficiency of different materials. In addition, the continuous electron beam scanned the sample while synchronously recording the CL spectra to produce CL mapping images by the CCD. When the sample was excited using the fs electron pulses with an electron energy of 30 keV induced by the UV fs laser pulses, the transient CL signal was generated and delivered to the spectrometer. The transient CL signal was then registered using a streak camera (with a resolution of ≈2 ps) to obtain hyperspectral TRCL images. The temperature‐dependent time‐integrated CL and TRCL measurements were conducted on a cryogenic cooling stage integrated inside the SEM. Note that the sample was kept stationary during the measurements, and the SUEM imaging and CL/TRCL measurements were carried out on the same microscopic area of the sample.

## Conflict of Interest

The authors declare no conflict of interest.

## Author Contributions

Y.Z. and X.C. contributed equally to this work. X.F. conceived the research project. X.F. designed and constructed instrument with input from Y.Z., X.C., Y.Y., Y.H., and F.L. Y.Z. and X.C. performed the experiments. Y.Z., X.C., and Y.Y. contributed to data analysis. Y.Z. and X.C. drafted the manuscript with input from X.F. All the authors contributed to the discussion and revision of the manuscript.

## Supporting information

Supporting Information

Supplemental Movie 1

Supplemental Movie 2

## Data Availability

The data that support the findings of this study are available from the corresponding author upon reasonable request.
