# Supplementary material for: A Femtosecond Electron‐Based Versatile Microscopy for Visualizing Carrier Dynamics in Semiconductors Across Spatiotemporal and Energetic Domains
Source: Adv Sci (Weinh). 2024 Jun 18;11(31):2400633. doi: 10.1002/advs.202400633 (PMC11336951; doi:10.1002/advs.202400633)
Supplement: Supplementary file 1 — Supporting Information [file ADVS-11-2400633-s003.docx]

Supplementary Information for

A femtosecond electron-based versatile microscopy for visualizing carrier dynamics in semiconductors across spatiotemporal and energetic domains

**Authors:** Yaqing Zhang^1#^, Xiang Chen^1#^, Yaocheng Yu^1^, Yue Huang^1^, Moxi Qiu^1^, Fang Liu^1^, Min Feng^1^, Cuntao Gao^1^, Shibing Deng^1^, Xuewen Fu^1,2*^

**Affiliations:**

^1^Ultrafast Electron Microscopy Laboratory, The MOE Key Laboratory of Weak-Light Nonlinear Photonics, School of Physics, Nankai University, Tianjin 300071, China

^2^School of Materials Science and Engineering, Smart Sensing Interdisciplinary Science Center, Nankai University, Tianjin 300350, China

#*Yaqing Zhang and Xiang Chen contributed equally.*

*Corresponding authors:

xwfu@nankai.edu.cn (X. F.).

**
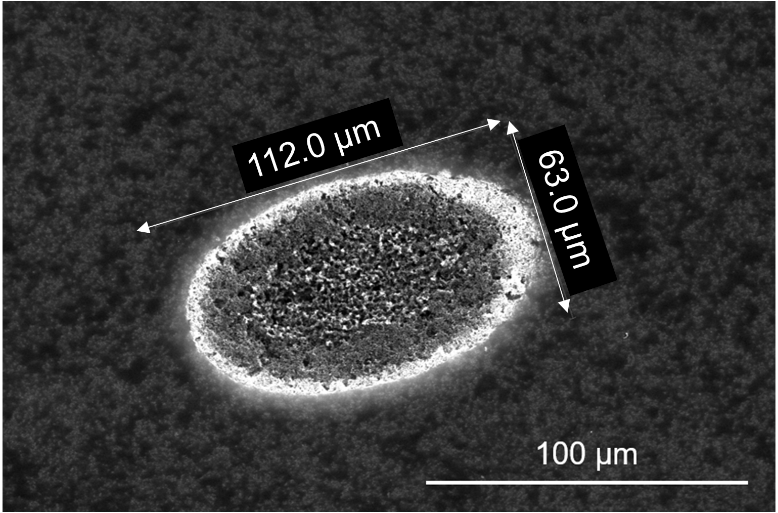
**

**Figure S1. The SEM image of the pump laser spot (power of ~70 mW) ablated on a carbon film.** The long and short axes of the pump laser spot on the sample are ~112 μm and ~63 μm, respectively.


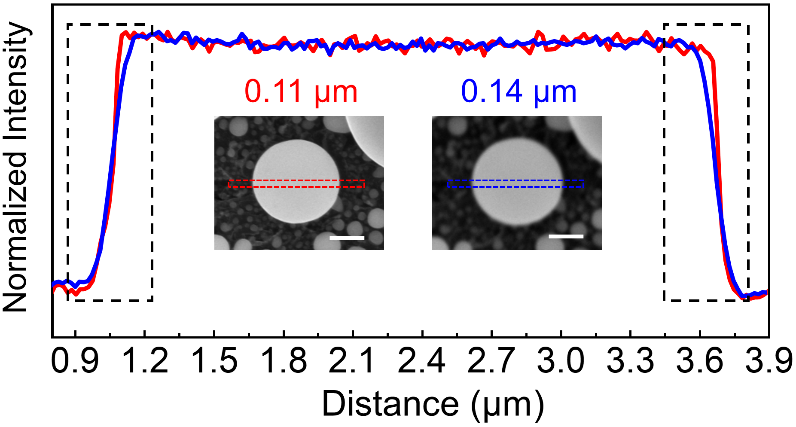


**Figure S2. The intensity profile analysis of the same tin sphere in the SEM images under field emission and photoemission modes.** Dashed black boxes indicate the edge of the tin sphere, and the rising widths (from 10% to 90% of the intensity) at the edges are 0.11 μm and 0.14 μm for the field emission continuous electron mode (red) and photoemission pulsed electron mode (blue), respectively. All scale bars are 1 μm.


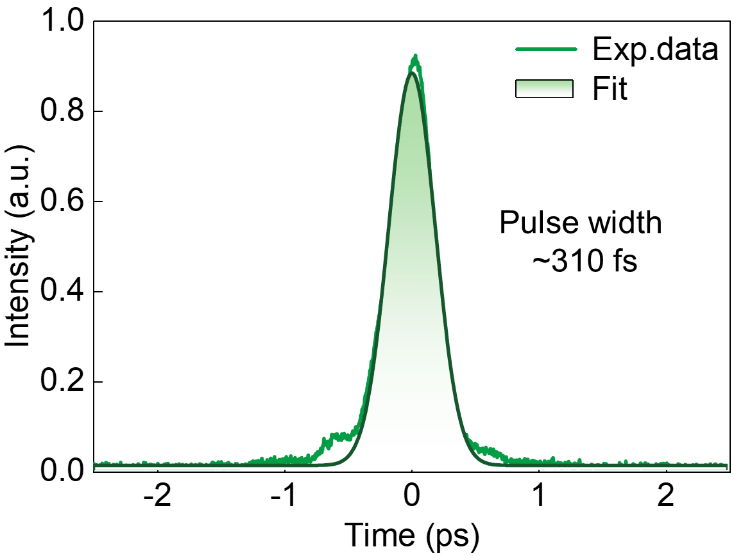


**Figure S3. Temporal profile of the pump laser pulse (515 nm) measured by an autocorrelator.** The temporal profile of the second harmonic laser was fitted by a Sech2 function, and the pulse duration was determined to be ~310 fs.


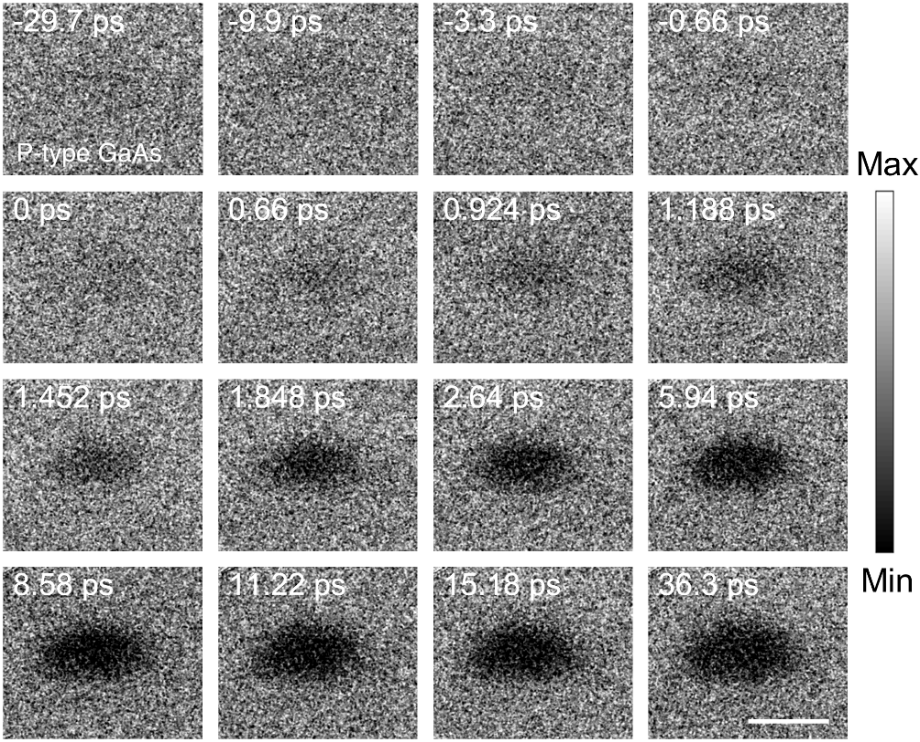


**Figure S4. The SUEM images of a p-type GaAs single crystal at a repetition rate of 5 MHz.** There is no obvious SUEM signal before time zero. Upon the laser pulse excitation, the dark contrast rapidly appears in the area of laser irradiation and slowly recovers with the delay time. The scale bar is 100 μm.


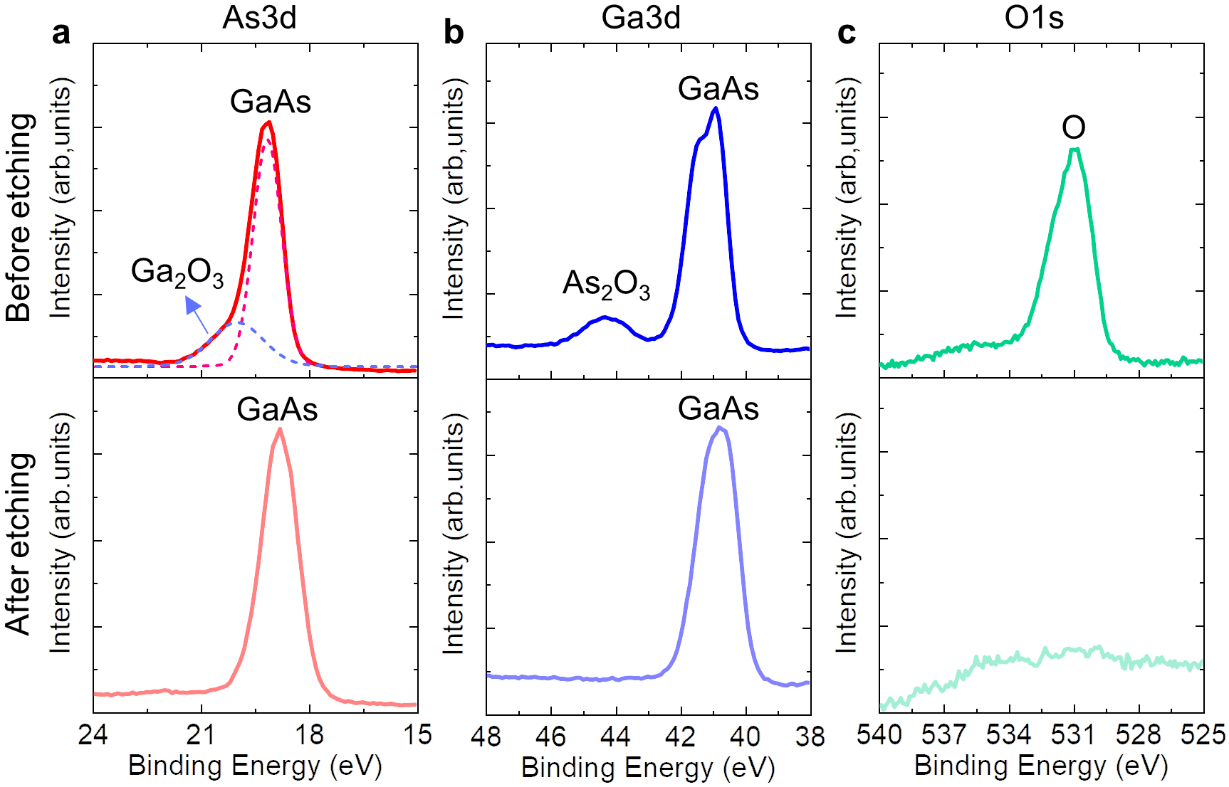


**Figure S5. The XPS spectra of GaAs (100) surface before and after Ar-ion etching.** The high-resolution XPS spectra of (a) As 3d. (b) Ga 3d. (c) O 1s.


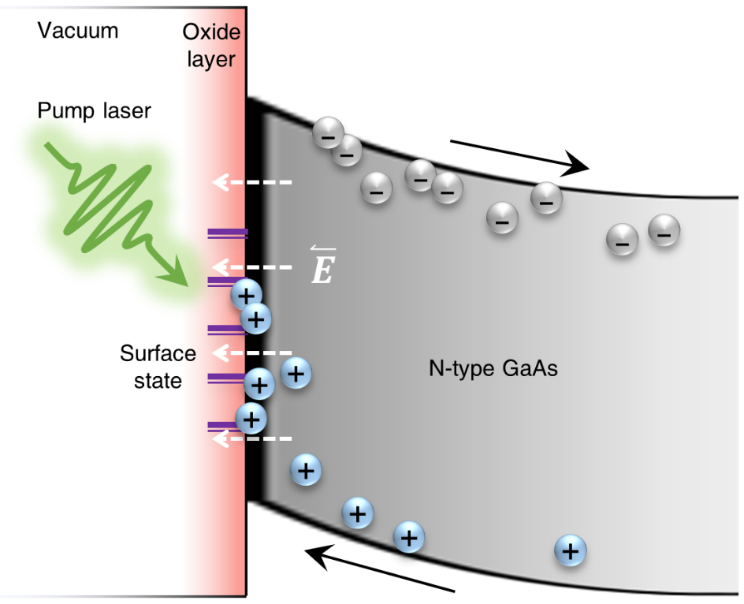


**Figure S6.** **Energy band diagram for the heavily silicon-doped n-type GaAs surface with a native oxide layer.** The oxide layer on the GaAs surface induces a high density of surface states, resulting in an upward bending of the surface energy band. Such upward bending of the surface energy band leads to a built-in electric field near the GaAs surface oriented from the interior to the vacuum. When the pump laser irradiates the GaAs surface, a large number of excess electrons (gray spheres) and holes (blue spheres) are generated inside and diffuse around. Because of the build-in electric field, the excess electrons will migrate to the bulk beneath the surface, while the excess holes will migrate to the surface and be trapped by the empty trapping or recombination centers (surface states), resulting in the dark contrast in the SUEM imaging result.


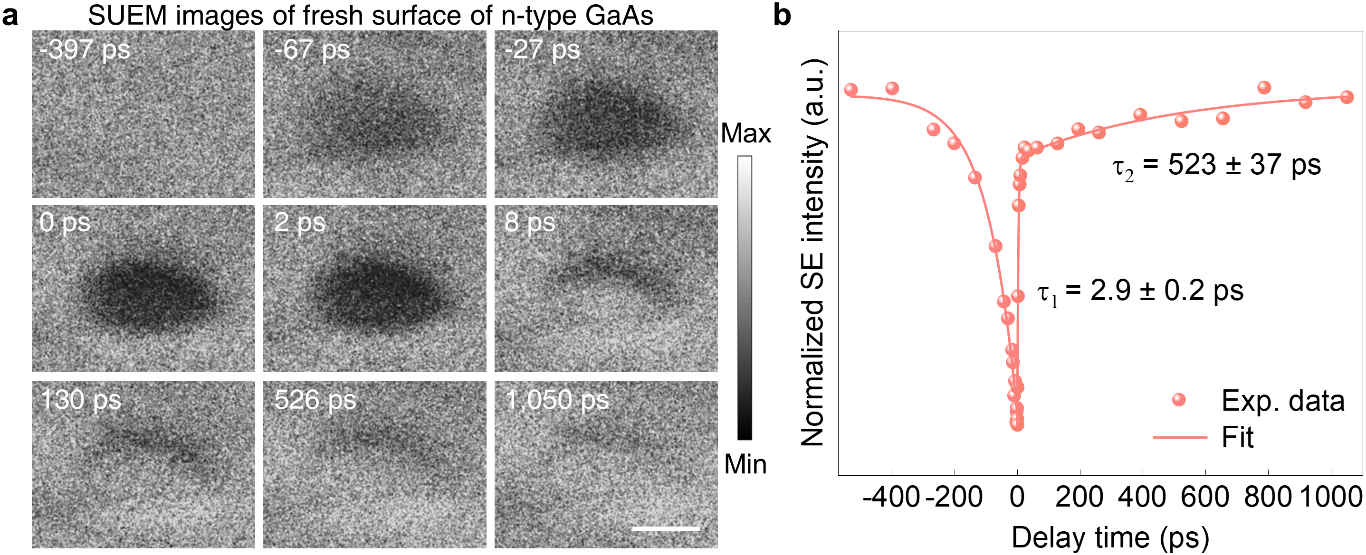


**Figure S7.** **SUEM imaging measurements on a fresh cleaved cross-section surface of the heavily silicon-doped n-type GaAs single crystal.** (a) SUEM images recorded at different delay times under a pump laser fluence of ~3.74 μJ/cm^2^ and repetition rate of 5 MHz (see also **Movie S2**). The scale bar is 50 μm. (b) The extracted dynamic curve from (a), reflecting the time-dependent SE intensity change at the center of the photo-excited region. The results show that the trend of the SUEM signal intensity at negative delay times is similar to that observed on the surface with a nature oxide layer presented in the main text. In contrast, following the laser pulse excitation, the dark contrast rapidly disappears in a very short time range, indicating there are much fewer trapping sates on the fresh cleaved cross-section surface of the n-type GaAs single crystal.

**
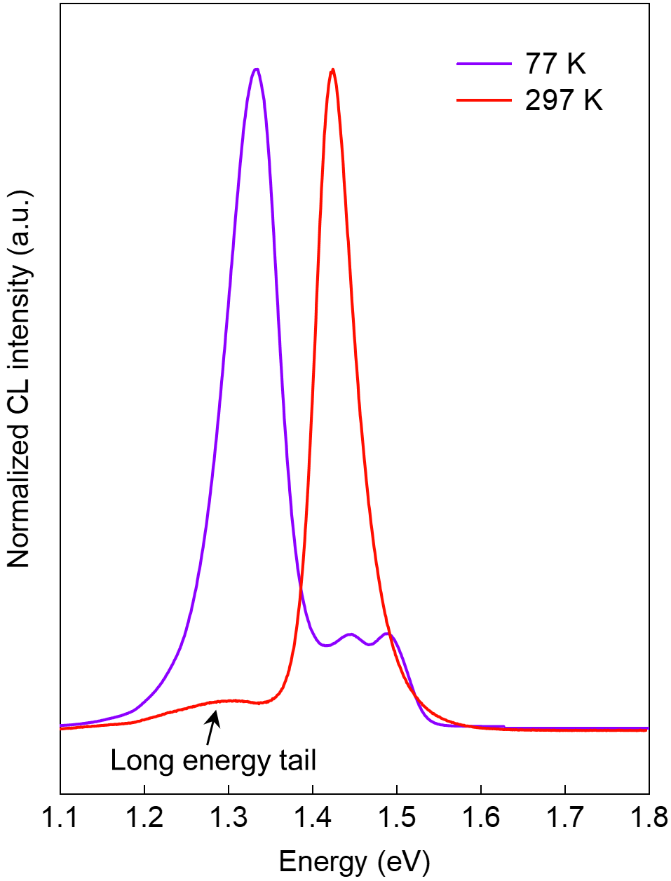
**

**Figure S8.** **Time-integrated CL spectra of the n-type GaAs single crystal at different temperatures.** Both of the data were recorded under the same primary photoemission pulsed electron excitation at an accelerating voltage of 30 kV. Note that there is a weak long energy tail at the low energy side of the CL spectrum at room temperature, indicating the presence of defect states in the n-type GaAs. As the temperature decreases from 297 to 77 K, the intensity of the long energy tail gradually increases.


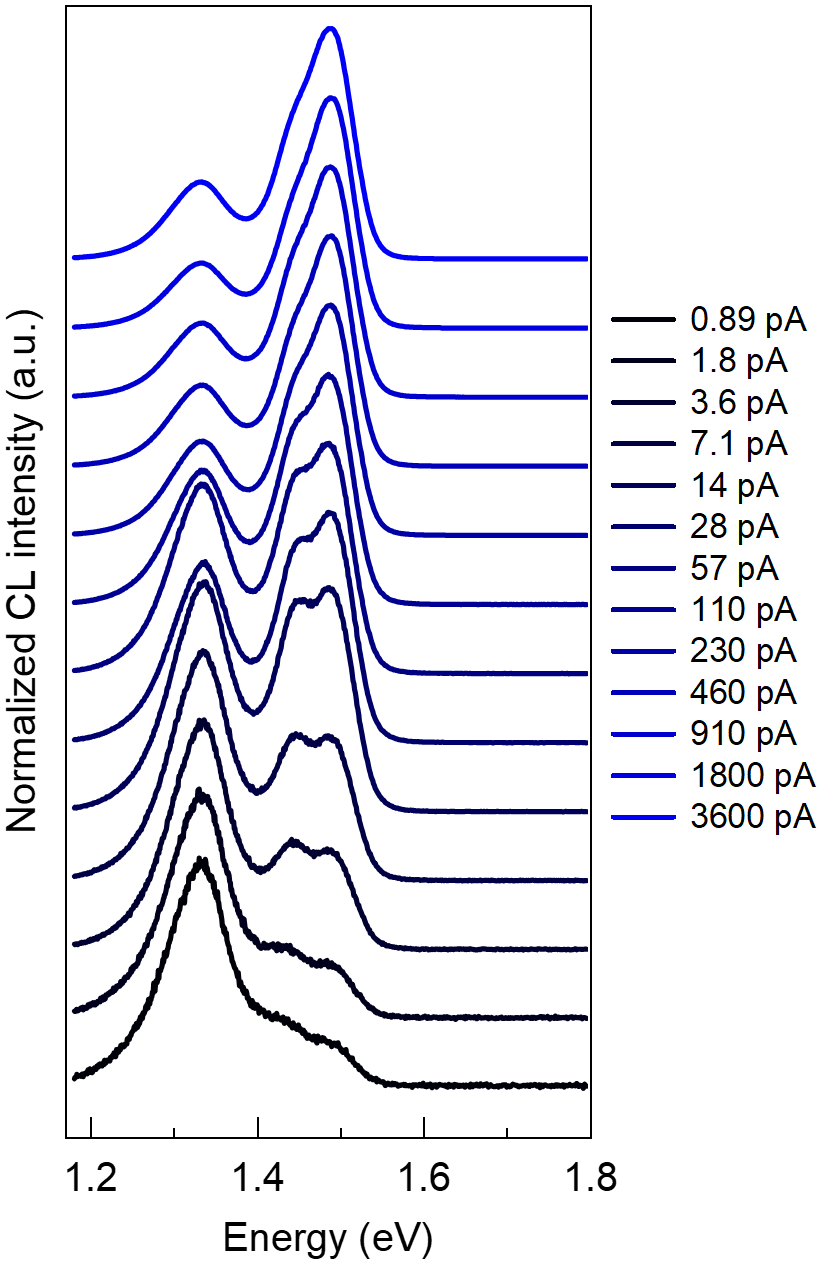


**Figure S9. Excitation beam current-dependent CL spectrum of the n-type GaAs single crystal.** All data were recorded under continuous electron excitation at an accelerating voltage of 2 kV and at a temperature of 77 K.


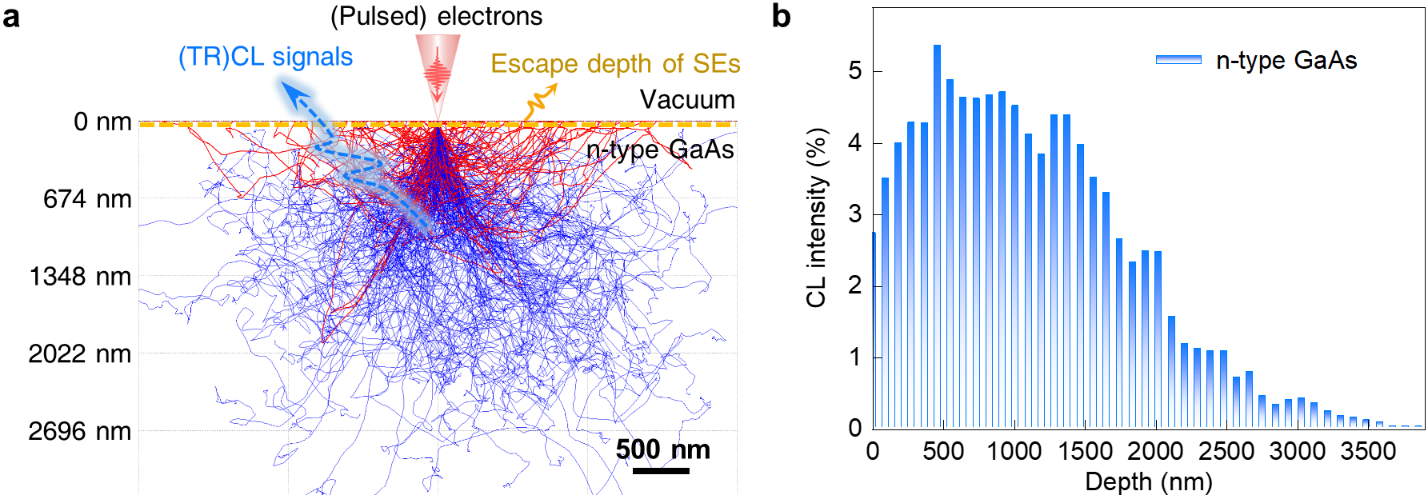


**Figure S10.** **Simulation of the interaction volume of electron scattering and CL production in the n-type GaAs single crystal at 297 K.** (a) Monte Carlo simulation (using the Casino software) of a 30 keV pulsed or continuous electron beam (containing ~300 electrons) impinging on the sample surface, which results in the emission of SEs and CL signals from the surface and inside. The internal electrons undergo elastic and inelastic collisions, forming a localized teardrop-shaped interaction volume beneath the surface, where the CL signals (blue dotted arrow) are generated and emit in the depth of micro scale, while only the SEs (solid yellow arrow) within the depth of a few nanometers below the surface (thick yellow dotted line) can escape and be collected by the ETD. The solid blue and red lines represent the trajectories (~1,000 tracks in total) of the SEs and backscatter electrons, respectively. (b) CL intensity corresponding to at different depths in (a). Clearly, the CL signals are mainly derived from the micro region of the bulk beneath the surface.
